# Reliability and validity of the Chinese revised version of the pectus excavatum evaluation questionnaire in children with pectus excavatum

**DOI:** 10.1186/s12887-024-04598-1

**Published:** 2024-02-19

**Authors:** Yujia Wu, Qi Zeng, Yaguang Peng, Chenghao Chen, Na Zhang, Jie Yu

**Affiliations:** 1 Department of Thoracic Surgery, Beijing Children’s Hospital, Capital Medical University, National Center for Children’s Health, No.56, Nan Li Shi Road, Xi Cheng District, Beijing, 100045 China; 2 Center for Clinical Epidemiology and Evidence-based Medcine, Beijing Children’s Hospital, Capital Medical University, National Center for Children’s Health, No.56, Nan Li Shi Road, Xi Cheng District, Beijing, 100045 China

**Keywords:** Pectus Excavatum evaluation questionnaire, Health-related quality of life, Reliability test, Validity test

## Abstract

**Objective:**

This study aims to translate the Pectus Excavatum Evaluation Questionnaire(PEEQ) into Chinese, and to comprehensively assess subjective outcomes in quality of life of children with pectus excavatum.

**Methods:**

The PEEQ was translated from English to Chinese as according to the PRO translation guidelines. Structural validity and reliability of the questionnaire were examined by validated factor analysis and Cronbach’s alpha coefficient analysis respectively.

**Results:**

The results of the validation factor analysis for the Chinese PEEQ parent’s and child’s questionnaires demonstrated that the fit indicators for each dimension met the required criteria. The overall Cronbach’s alpha coefficient of parent’s and child’s questionnaires were 0.840 and 0.854. Both the item-level content validity index (I-CVI) and scale-level content validity index (S-CVI) of each sub-questionnaire were 1.

**Conclusion:**

The Chinese version of the PEEQ parent’s questionnaire is suitable as a proxy assessment for patients with PE, but the child’s questionnaire needs further adjustments.

## Introduction

There are various types of chest wall deformities, of which pectus excavatum(PE) and pectus carinatum(PC) are the two most common [1]. Pectus excavatum is a funnel-shaped deformity formed when part of the sternum, ribs and costal cartilages are concave toward the spine. The sunken bones compress the lungs and heart in the chest cavity, thus limiting cardiorespiratory function, decreasing exercise endurance, and partially accompanied by chest pain and pressure sensation, limiting the patient’s physical activity and decreasing the quality of life. The severity of chest wall deformity in children with PE tends to worsen during adolescence due to the significant physical and psychological changes that occur during this particular phase of body development. The majority of patients with PE experience significant body image concerns, leading to varying degrees of impact on their self-esteem and social life [2]. Consequently, physicians are increasingly focusing on finding effective methods to assess the impact of PE on the lives of affected children.

Health-related quality of life (HRQOL) refers to an individual’s subjective assessment of various health aspects, including the evaluation of physical activity function, mental health, social adjustment, as well as patient’s perceptions of his/her own life and health. Currently, HRQOL serves as a crucial tool for evaluating medical and public health interventions [3]. Body image and self-esteem are important factors in the evaluation of both quality of life (QOL) and health-related quality of life (HRQOL). Impairment of HRQOL due to illness may affect the patient’s perception of the outcome of treatment, which can lead to disagreement with the physician [4]. Thus, patients’ participations in such assessments serve as significant complements to physician opinions and physiological evidence.

The Pectus Excavatum Evaluation Questionnaire (PEEQ) has emerged as the primary assessment tool for evaluation of HRQOL of children with chest wall deformities. The PEEQ, devised by Lawson and his team in 2003, and they completed reliability and validity testing of the PEEQ [1]. is a telephone questionnaire for children between ages 8 and 13. It consists of two sets of sub-questionnaires to separately evaluate the HRQOL for the patient from both the child’s and their parents’ perspectives. The child’s questionnaire consists of 12 questions, divided into two dimensions: psychological and physical. The parent’s questionnaire consists of 13 questions, divided into four dimensions: psychological, physical, self-awareness, and guardianship concerns. The PEEQ investigates the degree of specific sensations or the frequency of specific behaviors of the patient in the last month [5], and can be applied to children before and after corrective surgery [1]. The PEEQ and its adult version, the NUSS assessment questionnaire (NQ-mA), modified by Krasopoulos and his team [6], have been widely used in clinical studies related to PE [5, 7–10]. They are commonly accepted as assessment tools for evaluating QOL of patients with PE.

Yet, studies in China on QOL of children with PE still lack a standardized tool, as there is no existing Chinese version of the PEEQ. To standardize an assessment of QOL of children in China, it is necessary to develop a tool applicable to Chinese children with PE. Therefore, the purpose of this study was to translate the PEEQ into Chinese, and to verify its validity as well as reliability within the cultural context.

## Methods

### Patient and study center

This study was a single-center study. Children with PE who visited the outpatient clinic of thoracic surgery and were hospitalized at the Department of thoracic surgery at Beijing Children’s Hospital, Capital Medical University from July 2021 to February 2022 were included as study subjects.

Inclusion criteria: (1) children between the ages of 8 and 18; (2) children diagnosed with chest wall deformity by thoracic surgeons through physical examination and ancillary tests; (3) children with PE who were not treated with surgical procedures or braces. Exclusion criteria: (1) children with other deformities or secondary chest wall deformities that are caused by other conditions; (2) children with conditions that may potentially affect their mental capacity.

This study was approved by the relevant expert committee and the ethics committee of Beijing Children’s Hospital of Capital Medical University([2022]-E-020-Y). The patients and their legal guardians were fully informed of the risks and signed informed consent forms before the clinical case studies were conducted.

### Translation process

**Fig. 1:**
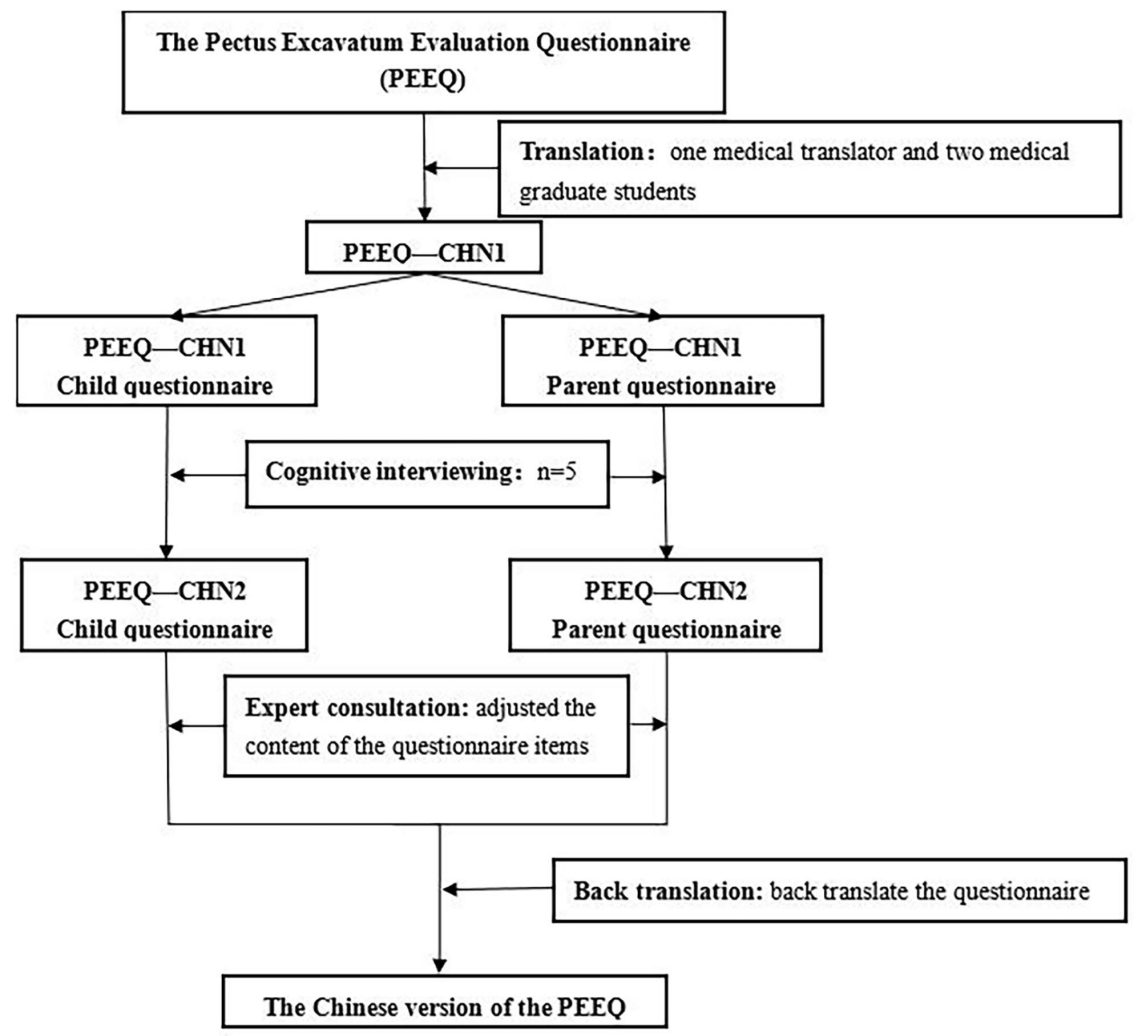
Study design for the translation process of PEEQ

Members of this research team contacted the developers of the original PEEQ questionnaire by email and obtained authorization. Referring to ISPOR’s Principles of Good Practice [11] and relevant literature [12], this study divided the translation and development processes into five main steps, including translation, proofreading, cognitive interview, expert consultation, and back translation. The specific steps are shown in Fig. 1.

### Validation process

#### Questionnaire survey

The finalized Chinese version of the PEEQ questionnaire was presented as a self-administered paper form. The Chinese version applies to children with chest wall deformities aged between 8 and 18. It is divided into two sub-questionnaires, one for the child’s self-assessment and the other for the parent’s assessment of the child. The parent’s and child’s questionnaires are divided into the same dimensions and retained the same scoring system as the original PEEQ [5].

Existing studies usually acknowledge that children aged 8 and above are able to assess their own HRQOL, as they are experiencing vital body development both psychologically and physically [13]. Therefore, questionnaires were completed by both the children and their parents independently.

During the translation phase of the questionnaire, five children were selected for interviews and it was determined that the children could understand and complete the questionnaire independently. The questionnaires were distributed to the children and their parents electronically. Detailed verbal instructions were provided to the participants while filling the questionnaires for quality control. Also, to ensure that each questionnaire was completed by the appropriate population, all questionnaires were completed in the presence of a physician. Training on standard procedures for questionnaire recording was conducted. Each questionnaire was transcribed promptly.

### Statistical analysis

Excel version 21.0 software was used for data entry. IBM SPSS Statistics version 26.0 and SPSS Amos Graphics CLI version 21.0 were used for data analysis. The frequency distribution of the total scores of the Chinese version was examined by graphical methods and normality tests. The bell-shaped distribution conformed to the normal probability curve in the histogram, and *p* > 0.05 in the Shapiro-Wilk normality test indicated that the data conformed to a normal distribution [12].

Based on relevant literature [14] and the actual situation, the sample size of this study was determined by a 5:1 ratio of subjects to questionnaire entries.

The adequacy of sampling was confirmed by the Kaiser-Meyer-Olkin (KMO) test and Bartlett’s sphericity test before performing structural tests. KMO value ≥ 0.70 and *p* < 0.05 in Bartlett’s test indicated that the data were suitable for factor analysis.

The content validity of the Chinese version was assessed through expert panel assessment. As required, the number of experts in the expert assessment process should be greater than 3 and less than 10 [15]. Therefore, we enrolled 5 experts in total, including 1 expert in child psychology and 4 experts in pediatric thoracic surgery. Each item in the questionnaire was scored by the experts on a scale ranging from 1 to 4, higher values on the response scale indicate a greater reflection of the actual experience. The results were displayed by the item-level content validity index (I-CVI) and scale-level content validity index (S-CVI). CVI is the unanimous S-CVI, i.e., the proportion of entries with all experts scoring ≥ 3 to the total number of entries. Considering that when the number of experts is ≤ 5, it is required that the experts’ opinions are unanimous, so that I-CVI of each question item should be 1.

The Chinese version of the parent’s questionnaire and child’s questionnaire were both examined through fit indices via confirmatory factor analysis (CFA). Based on theoretical considerations and statistical instructions, the model was modified to obtain the fitted solutions. The cutoff levels of the fit indices were: Root Mean Square Error of Approximation (RMSEA) < 0.08; Goodness of Fit Index (GFI), Comparative Fit Index (CFI), and Non-standard Fit Index (NNFI), also known as Tuker-Lewis Index (TLI) > 0.9 [16]. When confronted with an unsatisfactory structural model fit index, the model is then corrected based on the underlying theoretical framework and the MI correction index, specifically the cardinality test and comparison of the AIC and BIC principles.

The Cronbach’s α coefficient serves as an indicator for the reliability of the Chinese version of the PEEQ, and the acceptable range was set at ≥ 0.70 [12].

The correlation between scores of the child’s questionnaire and the parent’s questionnaire was tested with Spearman’s correlation coefficient. Spearman’s correlation coefficient r ranged from − 1 to 1, with positive correlations for positive r values and negative correlations for negative r values. In this study, 0 < *r* < 0.4 was considered a weak correlation, 0.4 < *r* < 0.7 was considered a moderate correlation, and 0.7 < *r* < 1 was considered a strong correlation.

## Result

### Sample description

A total of 110 parent questionnaires and 70 child questionnaires were distributed in this study, and after the recovery of the questionnaires, the valid questionnaires were 101 parent questionnaires and 61 child questionnaires. The overall recovery rates of the parent and child questionnaires were 84.6% and 85.8%, respectively. Table 1 summarizes the detailed sample characteristics of the study participants.

**Table 1:** Patients Characteristics of the Validation Cohort

		Child Questionnaires(*n* = 61)	Parent Questionnaires(*n* = 101)
Sex, n(%)			
Male	51(83.6)		84(83.2)
female	10(16.4)		17(16.8)
Age, n(%)			
8–12	19(31.1)		36(35.6)
13–18	42(68.9)		65(64.4)

The first three items on the child’s questionnaire in the Chinese version were considered to be reverse questions and needed to be positive before further testing. The positive items were labeled as Q1’, Q2’, and Q3’.

Meanwhile, we conducted the Kaiser-Meyer-Olkin (KMO) test and Bartlett’s sphericity test to ascertain the appropriateness of sampling for the Chinese version of parent’s and child’s questionnaires. The KMO values of both sub-questionnaires were ≥ 0.70 (KMO = 0.845 for the parent volume and KMO = 0.774 for the child volume) and *p* < 0.05. The results of these tests indicated favorable suitability for factor analysis.

### Validity tests

#### Structural validity

For the Chinese version of child’s questionnaire, we constructed factor models and conducted rigorous factor analysis in line with the theoretical structure of the original questionnaire. The results revealed inadequate model fit (χ2/Δf = 2.82; RMSEA = 0.08; GFI = 0.73; NNFI(TLI) = 0.70; CFI = 0.75). Among the observed variables Q3’, Q4 The factor loadings with the latent variables were significantly less than 0.5. Results were not optimal for two questions in the questionnaire, which may be related to children not understanding the question properly. As a result, the questionnaire model was considered to be a poor fit, requiring further adjustments.

**Fig. 2:**
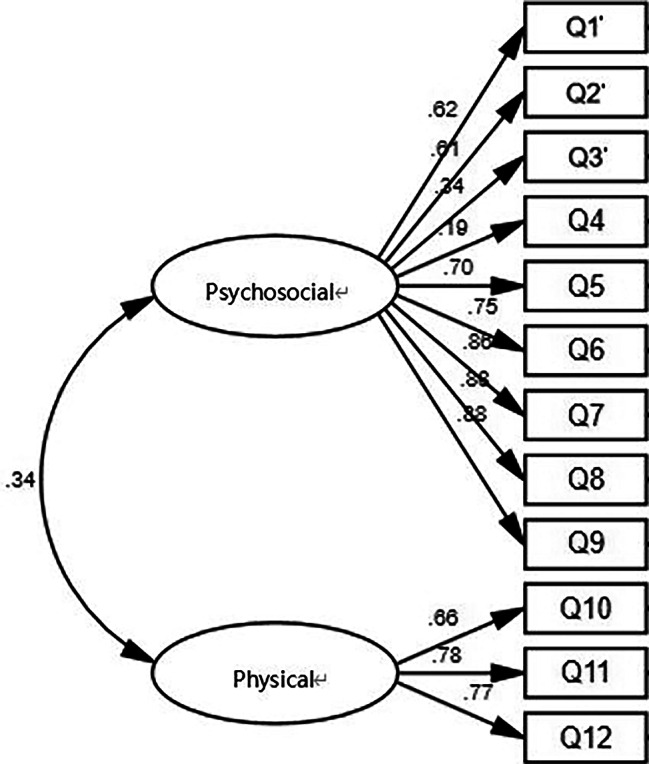
Model of child questionnaire

The average variance extracted (AVE) of the questionnaire was > 0.5, and the correlation coefficient between latent variables was significantly smaller than the square root of the AVE arithmetic. The structural model is shown in Fig. 2.

**Fig. 3:**
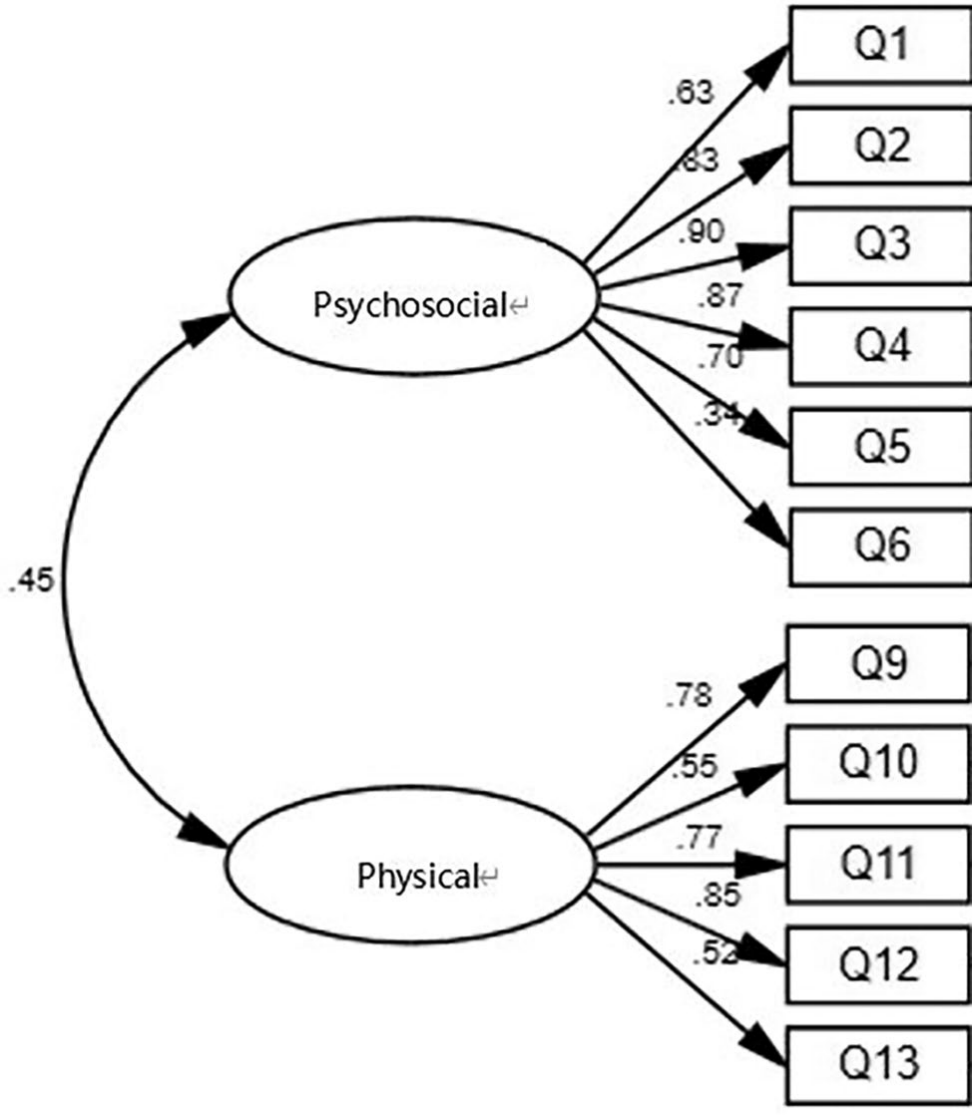
Model of parent questionnaire

After constructing a factor model for the parent’s questionnaire, a thorough factor analysis was also conducted. The results showed that the model fitted well (χ2/Δf = 1.35; RMSEA = 0.04; GFI = 0.91; NNFI(TLI) = 0.96; CFI = 0.97). Additionally, the questionnaire AVE > 0.5, and the correlation coefficient between latent variables was significantly smaller than the square root of AVE arithmetic. The structural model is shown in Fig. 3.

#### Content validity

Following statistical analysis, it was observed that the entry-level content validity indices (I-CVI) for both the parent’s and child’s questionnaires were 1.00. Also, the scale-level content validity index (S-CVI) reached a value of 1.00, which was greater than 0.80.

### Reliability tests

The Cronbach’s alpha of the child questionnaires was 0.854, and the Cronbach’s alpha of the parent questionnaires were 0.840. As shown in Tables 2 and 3, all subscales were above 0.70.

**Table 2:** Descriptive statistics of the individual items (child questionnaires)

Component (Cronbach’s α)	Question Number	Mean	Min-Max	Cronbach’s α
Psychosocial	Q1	2.55	1–4	0.874
	Q2	2.52	1–4	
	Q3	2.85	1–4	
	Q4	2.58	1–4	
	Q5	3.45	1–4	
	Q6	3.09	1–4	
	Q7	2.97	1–4	
	Q8	3.00	1–4	
	Q9	3.21	1–4	
Physical	Q10	3.45	1–4	0.778
	Q11	3.55	1–4	
	Q12	3.52	1–4	

**Table 3:** Descriptive statistics of the individual items (parent questionnaires)

Component (Cronbach’s α)	Question Number	Mean	Min-Max	Cronbach’s α
Psychosocial	Q1	3.11	1–4	0.869
	Q2	3.34	1–4	
	Q3	3.45	1–4	
	Q4	3.51	1–4	
	Q5	3.62	1–4	
	Q6	3.81	1–4	
Self-consciousness	Q7	3.70	1–4	-
Caregiver concern	Q8	-	-	-
Physical	Q9	3.55	1–4	0.787
	Q10	3.64	1–4	
	Q11	3.70	1–4	
	Q12	3.38	1–4	
	Q13	2.92	1–4	

### Questionnaire scores

The distribution of the total questionnaire scores was verified by histogram and Shapiro-Wilk D’Agostino-Pearson normality test. The results showed that the distribution of the total scores of the parent’s questionnaire was skewed (*p* < 0.05). On the other hand, the total scores of child’s questionnaire demonstrated a normal distribution (*p* > 0.05p = 0.18 > 0.05; *p* = 0.20 > 0.05).

#### Child’s and parent’s questionnaire scores by dimension

Since the number of questions within each dimension was different between the child’s and the parent’s questionnaire, a comparative analysis of the scores between the two was conducted by computing the mean score for each dimension. The mean score of each dimension was calculated by dividing the sum of the scores for each dimension by the number of items within that dimension. Specifically, for the psychological dimension, the mean score of items from the child’s questionnaire was 3.02, while the mean score of items from the parent’s questionnaire was 3.34. Moreover, for the physical dimension, the mean score of items from the child’s questionnaire was 3.32, while the mean score of items from the parent’s questionnaire was 3.06.

#### Correlations between child’s and parent’s questionnaires

Spearman’s correlation coefficients were used to examine the associations between scores of the child’s questionnaire and that of the parent’s questionnaire. The results indicated that the correlation coefficients for total scores ranged from 0.40 to 0.55 (*p*-values < 0.01), representing a weak to moderate positive correlation. Meanwhile, the correlation coefficients between scores on psychological dimensions were 0.34–0.45 (*p*-values < 0.01), representing a relatively weak correlation. However, the correlation coefficients between scores on the physical dimensions were 0.56–0.74 (*p*-values < 0.01), indicating a moderately strong positive correlation.

## Discussion

The PEEQ questionnaire is currently the most widely used type of assessment tool in studies related to chest wall deformities, providing a comparable quantitative evaluation index for different studies. Indications for treatment of patients with pectus excavatum include improvement of chest wall appearance in addition to clinical symptoms. Therefore the subjective evaluation of patients and their relatives is informative for treatment. For Chinese children with pectus excavatum, there is a lack of widely recognized assessment tools, and the results of related studies cannot be compared and generalized. The Chinese version of the PEEQ questionnaire can be used as an assessment tool, and become a reference index for comparisons between different studies. The PEEQ Chinese questionnaire can be used as an assessment tool and become a reference index for comparison between different studies. It can also provide subjective evaluation indexes from patients and their relatives for follow-up studies and provide reference data for clinical treatment.

The purpose of this study was to translate the PEEQ into Chinese and to assess the validity and reliability of the translated version. The results showed that the entire questionnaire had a good level of reliability, as indicated by Cronbach’s α. While there are no definitive guidelines for the translated versions of established measures in research, α ≥ 0.70 is commonly accepted as a reliable measurement in population studies [12].

The Chinese version of the child’s PEEQ did not align well with the original hypothetical structural model, as it failed to meet the required criteria. Specifically, the factor loadings between question 3 and question 4 and their respective latent variables were found to be less than 0.5. This suggests that these two questions did not adequately capture the psychological aspects of the child’s situation. To Question 3 (How would you feel if your chest looked like this in the future?), the children generally answered that they had never thought about this, or that they could not make a choice because they could not predict the future situation. To Question 4 (whether they were teased by other students), the children answered that they were able to avoid the deformity being seen by covering up their chest with clothing. It is also important to note that the situation described in this question might be ignored or deliberately avoided by the children. The CFA results of the Chinese version of the parent’s questionnaire demonstrated a good fit with the original hypothesized model, indicating that the parent’s questionnaire effectively reflected the situation of children with PE in China.

The correlation between the scores of the child’s questionnaire and the parent’s questionnaire was also examined. Comparing the scores of the child’s and their parent’s questionnaire, there was a moderate correlation between their total questionnaire scores, but a weak correlation in the psychological dimensions. This suggests that relying solely on parents’ assessments might not fully and objectively reflect the psychological impact of the deformity on children older than 8. This disparity might be related to the fact that adolescent children tend to be hesitant in communicating with their parents.

Furthermore, scores on the main dimensions of the child’s and the parent’s questionnaire were compared. Child’s self-assessment showed that the physical dimension was the least affected, whereas the parent assessment showed that the psychosocial dimension was the least affected. However, results from other studies on HRQOL typically exhibit a more substantial influence on the psychosocial aspects from the parent’s questionnaire, meaning lower scores in psychological dimensions [17]. This discrepancy between previous research and the outcomes presented in the present study merits further examination and warrants a careful interpretation of the results.

This study has several limitations. Firstly, this is a single-center study conducted exclusively at Beijing Children’s Hospital, Capital Medical University. As a result, there is a potential for selection bias in the choice of subjects. But our center is a national children’s medical center, and our patients come from all over the country to effectively represent the characteristics and basic conditions of children with pectus excavatum. Secondly, the retest reliability test was not conducted, highlighting a need for further improvements in this aspect.

## Conclusion

This study reveals that the reliability test of the Chinese version of the PEEQ was satisfactory. The Chinese version of the parent’s questionnaire was a suitable proxy assessment tool for patients with PE. However, some adjustments are required for the child’s questionnaire. The Chinese version can be used as an evaluation tool to collect standardized HRQOL data for children with PE both before and after treatment, making it a crucial measure for evaluating treatment effectiveness and has the potential to be widely adopted in clinical practice.

## Data Availability

The datasets used and/or analysed during the current study are available from the corresponding author on reasonable request.

## Abbreviations

PEEQ: The Pectus Excavatum Evaluation Questionnaire
QOL: quality of life
HRQOL: Health-related quality of life
NQ-mA: Nuss evaluation Questionnaire modified for Adults
SSQ: Single Step Questionnaire
PedsQL: The Pediatric Quality of Life Inventory Measurement Models
CFA: confirmatory factor analysis
EFA: exploratory factor analysis
I-CVI: item-level content validity index
S-CVI: scale-level content validity index
Cronbach’s α: Cronbach’s alpha coefficient
KMO: Kaiser-Meyer-Olkin
RMSEA: root mean square error of approximation
GFI: goodness-of-fit index
CFI: comparative fit index
NNFI: Non-Normed Fit Index
TLI: Tuker-Lewis index
AIC: Akaike information criterion
BIC: Bayesian Information Criterion
